# Correlation of Prolonged Sitting Time and Sitting Posture on Low Back Pain: A Cross-Sectional Study among Medical Students at Universitas Sumatera Utara

**DOI:** 10.12688/f1000research.146596.2

**Published:** 2026-09-15

**Authors:** Jocelyn Koswara, Yetty Machrina, Mahrani Lubis, Rina Amelia

**Affiliations:** 1 Medical Study Program, Faculty of Medicine, Universitas Sumatera Utara, Medan, North Sumatra, 20155, Indonesia; 2 Department of Physiology, Faculty of Medicine, Universitas Sumatera Utara, Medan, North Sumatra, 20155, Indonesia; 3 Department of Pediatrics, Faculty of Medicine, Universitas Sumatera Utara, Medan, North Sumatra, 20155, Indonesia; 4 Department of Community Medicine, Faculty of Medicine, Universitas Sumatera Utara, Medan, North Sumatra, 20155, Indonesia

**Keywords:** Low back pain, Medical students, Sitting posture, Sitting duration, Indonesia

## Abstract

**Background:**

Low Back Pain (LBP) is the most common musculoskeletal disorder in the world. Medical students, particularly those who use computers for study purposes, are more vulnerable to LBP due to prolonged sitting times and improper sitting posture during lectures. However, prior research showed that the prevalence of LBP among medical students appeared lower when they were enrolled during online pandemic learning. Hence, this research is performed to determine the correlation between the duration of sitting and sitting posture on the incidence of LBP among medical students in Universitas Sumatera Utara, Indonesia.

**Methods:**

A cross-sectional study with an analytical descriptive design was conducted in September 2023 among randomly selected 147 medical students at Universitas Sumatera Utara, Indonesia. The inclusion criteria of the research sample are students with BMI< 23 kg/m2 while the exclusion criteria are students who have abnormalities and a history of trauma or spinal fractures. Data was obtained from filling out questionnaires of The Pain and Distress Scale (PAD) and Body Awareness of Postural Habit in Young People (BAPHY). A Chi-square test and logistic regression were used to analyze data with a p-value < 0.05 was regarded as statistically significant.

**Results:**

The study’s findings revealed that 40.1% of students reported having LBP problems, 72.1% reported sitting for more than seven hours each day, and 33.3% reported having a bad sitting posture. Chi-square analysis revealed a significant correlation between sitting duration and posture on complaints of lower back pain (RR = 1.69, p = 0.041 and RR = 1.69, p = 0.009). At a risk of 2,438 times, sitting posture is the main factor contributing to LBP among medical students at Universitas Sumatera Utara, Indonesia.

**Conclusions:**

This research showed a significant correlation between duration and sitting posture on the incidence of low back pain.

## Introduction

Low back pain (LBP) is the most prevalent musculoskeletal condition in the world, affecting 15–45% of people annually. Approximately 17.3 million individuals in the UK have suffered from low back pain, leaving 1.1 million immobilized. Additionally, it was reported that 26% of American adults complained about having low back discomfort at least one day every three months. According to National Basic Health Research of Indonesia (RISKESDAS) 2018, the prevalence of low back pain in Indonesia is expected to be between 7.6% and 37% and will continue to increase as people age. ^1^

Most cases of LBP have a multifactorial etiology. ^2^ The two most common factors associated with LBP complaints are extended periods spent sitting and improper posture. ^3^ Up to 60% of adults report having low back pain because of sitting issues. Additionally, according to WHO, prolonged sitting can result in musculoskeletal issues, which can ultimately cause LBP. ^4^

The risk of low back pain (LBP) can be elevated by prolonged sitting without a break. This is because sitting increases the pressure on the spine by two times, which can lead to an increase in biomechanical processes that can alter the form of the spine. ^5^ According to Wibowo, ^6^ an individual’s risk of low back pain (LBP) can be increased by 2.68 times by adopting an incorrect sitting posture, such as bending over, as opposed to sitting erect. The reason behind this is that a hunched sitting position can impose prolonged strain on the body’s muscles, making it an awkward and uncomfortable working posture.

Medical students are particularly vulnerable to LBP because of several risk factors, including stress, long study sessions, improper sitting postures, and insufficient physical activity. ^2^ Suffering from low back discomfort might hinder students’ performance and affect their education starting from the pre-clinical period and ultimately throughout their medical career. ^7^

According to Amelot *et al*., ^8^ medical students usually study for more than seven hours every day. The rigorous lecture schedule forces students to sit still for extended periods, leading many of them to adopt incorrect sitting postures that increase their risk of developing musculoskeletal conditions like LBP. ^9^ Medical students in Universitas Sumatera Utara (USU) engage in instructional activities on campus for roughly seven to nine hours per day. These kinds of activities include open lectures, tutorials (group discussions), laboratory practicums, and clinical skills labs that require students to sit longer. Moreover, most of them who frequently use computers in class while sitting with poor posture will also increase the likelihood of developing LBP. ^10^

According to research by Vujcic et al. ^11^ in Belgrade (Serbia), the prevalence of medical students who experienced LBP was 59.5%. In France, the prevalence of LBP experienced by medical students was 72.1%. ^8^ Meanwhile in Indonesia, the prevalence of research results conducted by Mutiara at Sriwijaya University among medical students regarding long relationships and sitting positions on LBP complaints was 59.4%. ^12^ However, research undertaken during the COVID-19 pandemic revealed that the frequency of LBP among medical students at USU was lower than other research, at 29,1%. Hasibuan claimed that a variety of factors, including the sitting position at home and the design of the chairs and tables used, affect the low prevalence which is difficult to evaluate in her research. ^13^

Based on previous studies, a preliminary survey was conducted among twenty medical students at USU about LBP. Of the twenty students, it was discovered that eighteen of them complained of LBP anytime between or after lectures. Therefore, it is important to analyze whether there is a relationship between duration and sitting posture during lectures on LBP complaints among medical students at Universitas Sumatera Utara (USU).

## Methods

### Study settings and population

This cross-sectional study used a descriptive-analytic methodology to analyze the relationship between prolonged sitting time and sitting posture on low back pain complaints among medical students at Universitas Sumatera Utara (USU). The study was conducted from September to December 2023 with a total of 147 out of 192 respondents selected by simple random sampling who met the inclusion and exclusion criteria. A required sample of 147 participants was determined using the cross-sectional study sample size formula as shown below, using an expected prevalence of 50%, a 95% confidence level, and a 5% margin.

$$Z_{1-\alpha /2}^{2}\times p(1-p)\times N$$

$$n=d^{2}(N-1)+Z_{1-\alpha /2}^{2}\times p(1-p)$$

### Inclusion and exclusion criteria

Full-time medical students studying at Universitas Sumatera Utara with a BMI below 23 kg m ^-2^ were included in the study. Students with anomalies and a history of trauma or spinal fractures were excluded.

### Instruments

Data was collected online using Google Forms by filling out The Pain and Distress Scale (PAD) and Body Awareness of Postural Habits in Young People (BAPHY) questionnaires which were all validated and used in previous studies and standardized by the World Health Organization and usage permission of the Indonesian version of the questionnaire has been asked from authors through direct messages. The questionnaire was divided into three sections which were all written in Indonesian.

The first section contained twelve questions related to socio-demographic data, history of fracture and spinal disorder, and sitting duration in one day throughout lectures. The second section was The Pain and Distress Scale (William J.K.Zung) questionnaire which in the Primala study consists of 20 questions about low back pain complaints in various conditions. The third section was the Body Awareness in Postural Habits in Young People (BAPHY) questionnaire consists of 8 questions about types of postural sitting habits that occupied during class.

### Data collection

Participants were assured to have informed consent before any data was collected and they were free to refuse or withdraw anytime in completing the questionnaire. The questionnaire was completed online via a Google form scanned from the given QR code and responses were saved through Google Drive.

### Data analysis

After receiving responses from participants, the selected sample’s data was analyzed using Statistical Product and Service Solution (SPSS) Version 27 Statistical Product a. Descriptive statistics were calculated and summarized in frequency and percentage. Bivariate analysis using the Chi-square test was to evaluate the association between sitting duration and posture toward LBP complaints. Multivariate analysis by logistic regression was performed to determine the most contributing factor in LBP. All statistical analysis was at a 5% level of significance (p < 0.05).

## Results

### Characteristics of participants

Among 147 selected medical students, 30.6% were male, and 69,4% were female. There are five age categories among the research samples, ranging from 18 to 22 with most of them being 20 years old (49%). Based on sitting duration, a total of 102 respondents (72.1%) claimed to sit more than seven hours each day during lectures. More than half of the respondents also exhibited a good sitting posture during lectures (66.7%) as data was taken from the BAPHY questionnaire. While PAD questionnaire showed that 47 participants reported having LBP, whereas the remaining participants said they had no LBP during lectures ( Table 1).

**Table 1.** Characteristic of participants.

Variables		Frequency (n=147)	Percentage (%)
**Gender**	Male	45	30.6
	Female	102	69.4
**Age group (Years)**	18	5	3.4
	19	47	32
	20	72	49
	21	22	15
	22	1	7
**Sitting duration (hours/day)**	>7	106	72.1
	≤ 7	41	27.9
**Sitting posture**	Good	98	66.7
	Bad	49	33.3
**LBP complaints**	Yes	59	40.1
	No	88	59.9

### Distribution of sitting posture

Most medical students at Universitas Sumatera Utara claimed that they often sit with their legs crossed, with the percentage being 49%. Most students stated that they always and frequently sat with both feet resting on the floor with a percentage of 34% and 34.7%, and only a few (4.8%) said they never sat with their feet resting on the floor. The percentage of students who always sat with their backs resting well on the backrest was 30.6%, while only 3.4% of students said they never sat with their backs resting on the backrests. As many as 14.3% of students stated that they never sat with their bodies pushed forward and students who always sat with their upper bodies twisted and their buttocks pushed forward were only 4.1% and 6.1% ( Table 2).

**Table 2.** Percentage distribution of students' sitting posture during lectures.

	Always (%)	Frequently (%)	Rarely (%)	Never (%)	Don’t know/Don’t remember (%)
Sit with your back well supported on the backrest	30.6	40.8	17.7	3.4	7.5
Sit with your body tilted forward	10.9	38.8	29.9	14.3	6.1
Sit with your upper body twisted	4.1	19.7	40.1	28.6	7.5
Sit with your upper twisted (with torso torsion)	6.1	27.2	32.7	27.2	6.8
Sit with your buttocks slipping forward	10.9	34.7	33.3	12.9	8.2
Sit with both feet firmly on the floor	34	34.7	21.8	4.8	4.8
Sit with your feet unsupported	4.8	22.4	42.2	21.1	9.5
Sit cross-legged	17	49	25.9	6.1	2

### Correlation of sitting duration on LBP

Based on Table 3, the Chi-square analysis results were obtained with a p-value of 0.041 (p < 0.05), which means there is a significant relationship between the sitting duration variable and LBP complaints. Of the 106 students who sat >7 hours/day, there were 48 students (45.3%) experienced LBP, meanwhile of the 41 students who sat ≤7 hours/day, there were 11 students (26.8%) experienced LBP. This shows that LBP complaints are mostly experienced by students who sit >7 hours/day. For the relative risk value, the result was 1.69, which indicates that people who sit >7 hours/day will have a 1.69 times risk of experiencing LBP complaints.

**Table 3.** Correlation of sitting duration and LBP and relative risk.

Sitting duration	LBP complaints				Total		P-value	Relative risk
	Yes		No					
	n	%	n	%	n	%		
> 7 hours/day	48	45.3	58	54.7	106	100	0.041	1.69
≤ 7 hours/day	11	26.8	30	73.2	41	100		
Total	59	40.1	88	59.9	147	100		

### Correlation of sitting posture and LBP

Based on Table 4, the results of the Chi-square analysis were obtained with a p-value of 0.009 (p < 0.05), which indicates that there is a significant relationship between the sitting posture variable and LBP complaints. Of the 98 students who had good sitting posture, there were 32 students (32.7%) experienced LBP, and 49 students who sat with bad sitting posture, there were 27 students (55.1%) experienced LBP. This indicates that students with bad sitting posture experience most LBP complaints. For the relative risk value, the result was 1.69, which indicates that people who sit with bad posture have a 1.69 times risk of experiencing LBP complaints.

**Table 4.** Correlation of sitting posture and LBP.

Sitting posture	LBP complaints				Total		P-value	Relative risk
	Yes		No					
	n	%	n	%	n	%		
Good posture	32	32.7	66	67.3	98	100	0.009	1.69
Bad posture	27	55.1	22	44.9	49	100		
Total	59	40.1	88	59.9	147	100		

### Multivariate with logistic regression

As can be seen in Table 5, the regression variable (Exp B) with the largest coefficient value, sitting posture, has a value of 2.438 (95% CI: 1.196–4.973), indicating it as the most significant risk factor out of the two. This table shows that participants who sit with poor posture have a twofold increased risk of lower back pain.

**Table 5.** Multivariate analysis with logistic regression.

Variable	Significance	Exp B	95% Cl
Sitting duration	0.063	0.466	0.209 – 1.043
Sitting posture	0.014	2.438	1.196 – 4.973

## Discussion

With a prevalence of 59.9%, this study revealed that the majority of medical students at Universitas Sumatera Utara did not report having LBP problems. This is consistent with research done in 2021 by Hasibuan on USU FK students, where the majority of the sample (70.9%) did not experience LBP. ^13^ However, there is a difference in the research conducted by Safitri in 2021 that the majority of medical students experienced LBP with a prevalence of 61.8%. The reason for this discrepancy is most likely that students with a BMI of ≥ 23 kg m ^−2^ were not excluded from Safitri’s research, which could have contributed confounding variables to the analysis. ^7^ Research by Abdu et al. ^14^ indicates that an individual who is overweight is more likely to get low back pain (LBP) because excess fat builds up in the abdominal region, which can weaken the abdominal muscles and tense the back muscles. Furthermore, the fact that women aged 20 years or younger made up most of the research sample may have contributed to disparities in prevalence. Therefore, there is still very little chance that LBP is the result of hormone imbalances. Studies conducted by Rasyidah et al. ^15^ have shown that fluctuations in estrogen levels can affect LBP complaints in women with hormonal disorders, including those who are menopausal, pregnant, or use contraception.

Medical students at Universitas Sumatera Utara (USU) attend lectures from 7 a.m. to 4 p.m. on a demanding schedule. Students who attend dense lectures must remain still for extended periods, which might lead to LBP symptoms. It was discovered in this study that most students (72.1%) sat through lectures for more than seven hours a day. This is because apart from the busy lecture schedule, the percentage of attendance requirements for USU medical students is also high, such as 80% in general lectures and 100% in practicums, tutorials, and skills labs, where students need to sit down during these activities. Table 3 reveals that students who sat for more than seven hours per day had a higher percentage of LBP complaints (45.3%). The Chi-square analysis results indicate a significant correlation between sitting duration and LBP complaints, with a p-value of 0.041 (p < 0.05). The study’s findings are consistent with research by Eloi et al. ^2^ that shows that sitting for longer than seven hours a day can affect LBP complaints. Students who sit for longer than seven hours a day may experience worsening symptoms and functional changes in comparison to those who sit for less than seven hours, with a p-value of 0.02 (p < 0.05).

Long periods of sitting can affect LBP complaints because they induce continuous loading, which modifies the lumbar vertebral curvature. ^16^ A packed lecture schedule in a matter of hours may worsen intra-discal vertebral compression, which in turn may cause back pain. ^14^ In addition, prolonged sitting can strain the muscles and ligaments in the back, particularly the posterior longitudinal ligament, which is the thinnest ligament in the L2-L5 region and is linked to low back pain. ^13^ Extended periods of static muscle contractions can impair blood flow to the muscles, which can lead to fatty acid buildup as a result of impaired glucose metabolism. This procedure may result in discomfort including exhaustion and lower back pain, which may affect an individual’s productivity at work. ^14, 17^

Besides the amount of time spent sitting, sitting posture can also affect lower back pain (LBP) problems. At 66.7%, the majority of USU FK students in the class of 2021 had decent sitting posture. This may be a result of the student’s prior knowledge of the correct sitting technique, which was covered in the dermato-musculoskeletal lecture during the previous semester. According to Table 4, which examines the association between sitting posture and LBP complaints, students who have bad sitting posture are more likely to report having LBP, at a prevalence of 55.1%, than students who have good sitting posture, at 32.7%. Chi-square analysis in this study found a significant relationship between sitting posture during lectures and complaints of LBP with a p-value of 0.009 (p < 0.05). These results are in line with Desenia’s research in 2023 which stated that poor sitting posture can cause pain due to less physiological conditions in the vertebrae with the p-value obtained being 0.0001 (p < 0.05). ^18^

The chance of developing LBP can be raised by bad sitting posture. Based on Table 5, the multivariate analysis’s findings indicated that students who sat poorly were 2.438 times (95% CI: 1.196–4.973) more likely to have low back pain (LBP). The present findings are consistent with the study carried out by Abdu et al., ^14^ which found that sitting position had the highest risk of producing lower back pain (LBP) among the three variables examined. An improper sitting posture can result in spasms and excessive straining of the ligaments and muscles surrounding the spine, which can lead to lower back pain (LBP). ^18^ LBP is more likely to develop in students who frequently sit with their backs tilted forward, cross their legs, or are hunched over. This is because sitting in these postures can produce discomfort by applying pressure to the spinal cord and soft tissues. ^15^ According to research by Wijayanti et al. ^19^ pain occurs more often in bent and upright sitting postures. An upright sitting posture can increase the pressure on the nerve bed by 140%, meanwhile sitting bent over can cause an increase in pressure by 190%. ^20^ Apart from that, disorders can also occur in the erector spine muscles in the form of tension due to excessive contraction. ^19^^,^^21^ Sitting hunched over can also increase the risk of a herniated nucleus pulposus (HNP). LBP patients may experience pain that radiates to the affected spinal nerve segment when their spinal nerves are squeezed as a result of an intervertebral disc rupture brought on by elevated pressure from an improper sitting position. ^22^ People who cross their legs while sitting can experience asymmetry in the left and right sides of the body, as well as increased rotation of the pelvic and lumbar spine. This can cause the hip joints to flex, which increases the chance of developing scoliosis or other spinal deformities. ^23^

LBP issues may also be influenced by the way tables and chairs are used and designed. According to Abdul et al. ^14^ The presence of armrests and seat cushions may have an impact on the chair’s seat and backrest angles. A 92 cm high table and a chair with suitable foot support are the best settings for studying, according to research by Sambo. ^16^ In order to prevent weariness and soreness, the chair’s backrest is also helpful in holding and supporting the back region. This is because when you sit on your back, the weight of your body is concentrated on your spine, which reduces joint tension and promotes greater muscle and tendon relaxation. ^20^

According to research by Hadi and Hasmar, ^20^ several factors need to be considered when sitting to prevent lower back pain (LBP). It is recommended that you sit with your buttocks touching the chair and your shoulders resting against the backrest. Your legs shouldn’t be crossed, and your knees should be maintained by bending at the proper angle at or below your hips. Elbows and arms can be rested on the support of a chair or table and you need to keep your shoulders relaxed. Avoid sitting in the same posture for more than 30 minutes and do not sit in a hunched iposture. If the chair does not have lumbar support, try to keep the back in a curved lumbar curve.

### Strength and limitation

The strengths of this study include the use of validated and reliable questionnaires, namely The Pain and Distress Scale (PAD) and Body Awareness of Postural Habits in Young People (BAPHY), as well as the use of simple random sampling to minimize selection bias and improve sample representativeness. Furthermore, the study focused on a relatively homogeneous population and controlled several confounding factors through strict inclusion and exclusion criteria. However, I also acknowledge several limitations, including the cross-sectional design, which cannot establish causality, the use of self-reported online questionnaires that may introduce recall and response bias, the limited generalizability of the findings, as the study was conducted in a single institution and one academic batch only, and other potential factors that may influence sitting posture, sitting duration, and low back pain, such as gender, sleep deprivation, physical activity, and psychological conditions, were not examined in this study due to the limited time available. 

### Ethical considerations

This study was approved on 7 ^th^ July 2023 by the Health Research Ethical Committee of Universitas Sumatera Utara with approval number 566/KEPK/USU/2023 based on the Declaration of Helsinki ( https://www.wma.net/policies-post/wma-declaration-of-helsinki-ethical-principles-for-medical-research-involving-human-subjects/). Participants were informed that they were free to refuse or withdraw anytime in completing the questionnaire and provided informed consent verbally to the authors before any data was collected. Consent was documented by filling out the consent paper whether the participant agreed or refused to participate in the research. In Indonesia, a person is considered an adult if she/he/they attended a university and no further informed consent from parents and guardians was necessary which means they have responsible for their own life to enter adulthood. ^24^

## Data Availability

Figshare: Correlation of Prolonged Sitting Time and Sitting Posture on Low Back Pain: A Cross-Sectional Study among Medical Students at Universitas Sumatera Utara. https://doi.org/10.6084/m9.figshare.24907428 This research contains the following underlying data:
- Raw data.xlsx (raw data in English and Indonesian) - SPSS LBP.sav (statistical analysis) Figshare: Correlation of Prolonged Sitting Time and Sitting Posture on Low Back Pain: A Cross-Sectional Study among Medical Students at Universitas Sumatera Utara. https://doi.org/10.6084/m9.figshare.24907428 This research contains the following extended data:
- Informed Consent.pdf (in English) - Ethical clearance.pdf - Research Questionnaire.pdf (questionnaire in English and Indonesian) All data is made accessible in accordance with the Creative Commons Zero “No rights reserved” data waiver (CC0 1.0 Public domain dedication). This research article adapted reporting guidelines from the Medical Education Unit of Universitas Sumatera Utara.
