# Supplementary material for: Correlation of Prolonged Sitting Time and Sitting Posture on Low Back Pain: A Cross-Sectional Study among Medical Students at Universitas Sumatera Utara
Source: F1000Res. 2026 Sep 15;13:1379. Originally published 2024 Nov 18. [Version 2] doi: 10.12688/f1000research.146596.2 (PMC13627944; doi:10.12688/f1000research.146596.2)
Supplement: Peer review history

# Reviewer report: Reviewer response for version 2

**DOI:** 10.5256/f1000research.202011.r521364

**Published:** 2026-09-30

**Authors:** Denny Agustiningsih

**Affiliations:** 1 Universitas Gadjah Mada, Yogyakarta, Special Region of Yogyakarta, Indonesia

Agree for the revision.

Is the work clearly and accurately presented and does it cite the current literature?

Partly

If applicable, is the statistical analysis and its interpretation appropriate?

Yes

Are all the source data underlying the results available to ensure full reproducibility?

Yes

Is the study design appropriate and is the work technically sound?

Partly

Are the conclusions drawn adequately supported by the results?

Partly

Are sufficient details of methods and analysis provided to allow replication by others?

Yes

Reviewer Expertise:

exercise physiology

I confirm that I have read this submission and believe that I have an appropriate level of expertise to confirm that it is of an acceptable scientific standard.

# Reviewer report: Reviewer response for version 1

**DOI:** 10.5256/f1000research.160697.r341987

**Published:** 2026-05-07

**Authors:** Sri Sumartiningsih, Yulingga Nanda Hanief

**Affiliations:** 1 Universitas Negeri Semarang, Semarang, Central Java, Indonesia; 2 Sports Science, Universitas Negeri Malang (Ringgold ID: 106251), Malang, East Java, Indonesia

The study examines a clinically and educationally significant issue: the correlation between extended sitting duration, sitting posture, and low back pain (LBP) among medical students. The research question is clear, the methodology is mostly right for a cross-sectional design, and the statistical analysis is simple.

 Please add the formula for sample size justification, expected prevalence, confidence level, margin of error, and design effect.

 In the results, please add 95% CIs for all effect estimates. Replace "children" with "students/participants."

 In the Discussion, please add a structured "Strengths and Limitations" subsection addressing cross-sectional design, self-report bias, unmeasured confounders, and BMI restriction.

Is the work clearly and accurately presented and does it cite the current literature?

Yes

If applicable, is the statistical analysis and its interpretation appropriate?

Yes

Are all the source data underlying the results available to ensure full reproducibility?

Yes

Is the study design appropriate and is the work technically sound?

Yes

Are the conclusions drawn adequately supported by the results?

Yes

Are sufficient details of methods and analysis provided to allow replication by others?

Yes

Reviewer Expertise:

exercise physiology, injury prevention and rehabilitation, sports performance

We confirm that we have read this submission and believe that we have an appropriate level of expertise to confirm that it is of an acceptable scientific standard.

# Response

**Published:** 2026-05-15

**Authors:** Jocelyn Koswara

Thank you very much for your valuable time, careful review, and constructive comments on our manuscript. I sincerely appreciate your feedback and suggestions, which have helped improve the quality and clarity of our study.

 I acknowledge the typing error in the manuscript where the term “children” was mistakenly written instead of “students/ participants.” I appreciate your careful observation regarding this issue, and the error will be corrected accordingly in the revised version of the manuscript. I would also like to clarify that the sample size calculation in this study was determined using the cross-sectional study sample size formula.

 In addition, I have added the strengths and limitations of the study in the discussion section. The strengths of this study include the use of validated and reliable questionnaires, namely The Pain and Distress Scale (PAD) and Body Awareness of Postural Habits in Young People (BAPHY), as well as the use of simple random sampling to minimize selection bias and improve sample representativeness. Furthermore, the study focused on a relatively homogeneous population and controlled several confounding factors through strict inclusion and exclusion criteria.

 However, I also acknowledge several limitations, including the cross-sectional design, which cannot establish causality, the use of self-reported online questionnaires that may introduce recall and response bias, and the limited generalizability of the findings, as the study was conducted in a single institution and one academic batch only due to the limited time available. I greatly appreciate the reviewer’s insightful feedback, which has helped improve the quality of our manuscript.

# Reviewer report: Reviewer response for version 1

**DOI:** 10.5256/f1000research.160697.r341988

**Published:** 2026-05-07

**Authors:** Denny Agustiningsih

**Affiliations:** 1 Universitas Gadjah Mada, Yogyakarta, Special Region of Yogyakarta, Indonesia

The work is presented clearly, although the analysis in the discussion is not extensive or sufficiently sharp. This is probably due to the lack of citation of recent literature and the lack of comparison with research in other countries. As an example, I have embedded here 3 papers that can be used as additional references. The author can also read paper 4 as an additional reference to deepen and sharpen the analysis in the discussion.

 In my opinion, the research design is appropriate, but a more detailed explanation of the sample size is needed. Since there are certainly more than 500 medical students at USU, why were only 192 recruited? It would be better to explain how the sample was calculated and what challenges were encountered during recruitment.

 This conclusion is overly simplistic, failing to consider other factors that also influence LBP, as noted in other studies. For a more accurate conclusion, other factors that may influence the relationship between posture and sitting duration, such as gender, sleep deprivation, physical activity, and psychological conditions, should be included in the analysis.

Is the work clearly and accurately presented and does it cite the current literature?

Partly

If applicable, is the statistical analysis and its interpretation appropriate?

Yes

Are all the source data underlying the results available to ensure full reproducibility?

Yes

Is the study design appropriate and is the work technically sound?

Partly

Are the conclusions drawn adequately supported by the results?

Partly

Are sufficient details of methods and analysis provided to allow replication by others?

Yes

Reviewer Expertise:

exercise physiology

I confirm that I have read this submission and believe that I have an appropriate level of expertise to confirm that it is of an acceptable scientific standard, however I have significant reservations, as outlined above.

# Response

**Published:** 2026-05-16

**Authors:** Jocelyn Koswara

Thank you very much for your valuable time, thoughtful review, and constructive comments on the manuscript. I sincerely appreciate your valuable suggestions, which have helped to improve the quality of our study.

 Regarding the number of participants included in this study, I would like to clarify that the final total of 192 participants was based on the inclusion and exclusion criteria that had been determined previously in the study protocol. Only participants who fulfilled all eligibility criteria were included in the analysis. Freshman students were not recruited as study subjects because they are relatively younger, and based on the literature we reviewed, the prevalence and risk of low back pain in this age group are still considerably lower compared to older students. Therefore, I considered that including freshman students might affect the homogeneity and relevance of the study population.

 In addition, I have added several additional references to strengthen the manuscript as suggested. However, recommended references could not be found or accessed through the system/database. Therefore, I included other relevant and accessible references with similar scientific context to support the discussion and findings of the study.
